# St. Bartholomew's Hospital

**Published:** 1907-05-25

**Authors:** 


### ST. BARTHOLOMEW'S HOSPITAL.

### IMPROVED METHODS AND A PROGRESSIVE SPIRIT.

We congratulate the Treasurer, Lord Ludlow, and the Clerk, Mr. Hayes, upon the improved form and complete character of the Treasurer's Report for 1906, with the accounts made out upon the Uniform System. The changes introduced are excellent and exhibit a progressive spirit in the management which we heartily welcome. The changes which have been made under the present treasurer in regard to the details of the workings of the several departments of the Hospital and Convalescent Home have been great and important. The checks now exercised over the economical management through the House Committee, the Visiting Governors' Committee, the Finance Committee, and the Drugs and Instruments and Appliances Committee, must have tended greatly to improve the whole administration. It is further satisfactory that the Treasurer records his indebtedness and thanks to the Medical Council for much valuable advice and assistance. We welcome the name of Mrs. Alfred Willett among the new Governors, for her husband has rendered yeoman service for many years. For some reason which is not stated, the qualification of the Governors has been raised from a donation of £50 to £100.

The Special Appeal Fund for Re-building does not make material progress. During the year less than £1,400 has been contributed to this fund, and in three years the total sum raised by special appeal has only reached £118,371. In addition to this sum, £6,600 has been received for special objects, including the Pathological Block, the New Nurses' Home, and the publication of the "History of St. Bartholomew's Hospital" by Dr. Norman Moore.

The number of in-patients treated during the year has been just over 7,000. The daily average number of patients in the Hospital has been 556, the average residence of each patient being 26.6 days. The total number of new cases in the Casualty, Midwifery and twelve Special Departments for out-patients has been 126,021. Inquiries were made into the pecuniary circumstances of 8,673 of these patients, of whom 380, or less than 5 per cent., were considered to be unsuitable for gratuitous aid; 1,070 in-patients have been sent to the Convalescent Home at Swanley, and during seven months 92 children were sent to the Metropolitan Institution at Broadstairs, for which the Hospital paid one guinea each out of its Samaritan Fund; 416 patients were provided with artificial limbs or other surgical apparatus, 466 were provided with money, 193 with clothing, and 153 with both money and clothing. Seven persons received an average of £10 each from the "Prisca Coborn Fund for the Relief of Incurables." It is interesting to note that the average stay of each patient at the Convalescent Home was 19.24 days, whilst the average cost of each patient was 20s. 4¼d. per week, a reduction of 1s. 3¾d. per head per week as compared with 1905.

The income of the Hospital in 1906 shows an increase of nearly £4,300 and of this increase upwards of £1,500 is derived from donations and legacies. The ordinary expenditure shows an increase of nearly £500, and there was an excess of expenditure over income for the year of £2,638. This increase, the Treasurer states, is more than accounted for by items which are of the nature of capital, rather than annual charges. The Treasurer, on the advice of the Finance Committee, has re-invested nearly £25,000 formerly standing in Consols, in securities yielding a higher rate of interest. The new investments mainly consist of 3 per cent. Consolidated Stock of the London County Council. At the instance of the Medical Council, the Medical Staff has been augmented by the appointment of an Assistant Surgeon to the Aural Department and an Assistant Physician to the Department for the Diseases of Women.

The Treasurer is strongly of opinion that a new Nurses' Home ought to be provided. The present buildings are in no way adequate for their purposes; they provide scanty room for the nurses, and are situated in so many different parts of the Hospital, that it is difficult to supervise them with due efficiency, or to conduct them with proper economy. It is not a little remarkable that the sum of £1,500 has been collected by past and present nurses for the new Home, which ought to be put in hand without a moment's avoidable delay. Thirty additional nurses have been added to the Staff during 1906, who have enabled the hours of duty of both Day and Night Nurses to be materially lessened. It is satisfactory to be able to record that Miss Ellen M. Greenstreet and Miss Mary Davies, after 27 and 35 years' service respectively as Sisters, on attaining the age limit, have been granted pensions. Miss M. R. Fowler, as Superintendent of the Trained Nurses' Institution since 1886, has been granted a pension of £50 per annum, as a gratuity, payable out of the profits made by the Nursing Staff of this Institution.

The City Corporation has arranged the lease of a permanent site from the Governors for one of the police ambulances which have recently been instituted in the City. A Racket Court will shortly be provided for the Resident Medical Staff who have now the advantage of occupying their new quarters facing Giltspur Street. It is anticipated that the new Out-patients' and Special Departments will be finished and ready for use in the autumn. The new Pathological Block now in course of erection for certain special work will cost nearly £30,000. Owing to reforms instituted by Lord Ludlow, the rental of the Hospital properties has been increased by £1,279 a year, whilst a saving in the annual cost of managing these properties has resulted to the extent of £374. It may be gathered from this record of the work done in connection with St. Bartholomew's Hospital during 1906, that under Lord Ludlow's Treasurership great improvements have been introduced and increased efficiency. So St. Bartholomew's Hospital, which is the oldest British Hospital, is now in a fair way to increase its usefulness and popularity with the patients and the public.

